# Audit Regarding the Physical Health Workload for Doctors at an Older Adult Psychiatry Unit in Leeds

**DOI:** 10.1192/bjo.2024.608

**Published:** 2024-08-01

**Authors:** Eleanor Morris

**Affiliations:** Leeds and York Partnership NHS Foundation Trust, Leeds, United Kingdom

## Abstract

**Aims:**

The Royal College of Psychiatrists provides guidance regarding the experience of Foundation Doctors and Core Trainees, during their Psychiatry rotations. At The Mount, an Older Adult Mental Health unit in Leeds, it was observed by trainees that management of physical health conditions was occupying a large portion of their time.

Aims:

1. Measure how much time is spent on physical health activities, between Foundation Doctors and Core Trainees at The Mount.
2. Consider the impact of physical health workload on the doctors’ experience of Psychiatry.
3. Explore the mental health experience of doctors during this rotation.

**Methods:**

This Audit was conducted in three stages:

1. Anonymous collection of quantitative data regarding the proportion of time spent on physical health work. This data was collected for current doctors across all wards, for a two-week period in October 2023.
2. A focus group of junior and senior doctors, to consider onward actions.
3. An anonymous qualitative survey regarding mental health experiences and suggestions for improvement. This was circulated to any Foundation Doctor or Core Trainee who worked at The Mount in the past 12-months, via an online survey.

**Results:**

The quantitative survey showed that Foundation Doctors and Core Trainees at The Mount were spending at least half their time on physical health jobs, such as: clinical reviews, skills such as blood tests and ECGs, and referrals to other clinicians or specialties. This was considered unsurprising by doctors at all levels during the focus group.

The qualitative survey explored this further, with observations that doctors were sometimes unable to attend MDT meetings, tribunals or CPAs due to the high physical health workload. It was felt that senior staff were proactive in offering support, however trainees still felt that opportunities for mental health experience were limited.

Suggestions for improvement were made during the survey, including:

1. Increased input by senior medical staff, such as GPs or Geriatric Trainees.
2. Additional staff to support with upkeep of equipment, or skills such as phlebotomy.
3. Increased use of technology rather than paper charts.
4. Greater clarity regarding minimum staffing and whether locum doctors can be arranged to cover gaps.

**Conclusion:**

In summary, the physical health workload for Foundation Doctors and Core Trainees was noted to be significant and impacting their mental health experience. Following this Audit, consultants at The Mount will be meeting with Senior Leadership to discuss methods for improving the training experience.

